# A crowd clustering prediction and captioning technique for public health emergencies

**DOI:** 10.7717/peerj-cs.1283

**Published:** 2023-05-04

**Authors:** Xiaoling Zhou, Guiping Zhu

**Affiliations:** 1 School of Journalism and Communication, Nanjing Normal University, Nanjing, Jiangsu, China; 2 Nanjing Television Station, Nanjing, Jiangsu, China

**Keywords:** Public health emergencies, Crowd clustering prediction, News text collection, Social problem management, Scene captioning

## Abstract

The COVID-19 pandemic has come to the end. People have started to consider how quickly different industries can respond to disasters due to this public health emergency. The most noticeable aspect of the epidemic regarding news text generation and social issues is detecting and identifying abnormal crowd gatherings. We suggest a crowd clustering prediction and captioning technique based on a global neural network to detect and caption these scenes rapidly and effectively. We superimpose two long convolution lines for the residual structure, which may produce a broad sensing region and apply our model’s fewer parameters to ensure a wide sensing region, less computation, and increased efficiency of our method. After that, we can travel to the areas where people are congregating. So, to produce news material about the present occurrence, we suggest a double-LSTM model. We train and test our upgraded crowds-gathering model using the ShanghaiTech dataset and assess our captioning model on the MSCOCO dataset. The results of the experiment demonstrate that using our strategy can significantly increase the accuracy of the crowd clustering model, as well as minimize MAE and MSE. Our model can produce competitive results for scene captioning compared to previous approaches.

## Introduction

Research is underway on how people from all walks of life usually perform during public health emergencies. In the context of such events, gathering people produces the majority of the textual gathering of news material and societal concerns. As a result, we examine the characteristics of news reporting and social issues. According to the data, there is usually a sizable throng present when the news and social issues first surface. To collect news text about the abrupt public health incidents, we propose a prediction model of crowds gathering and social concerns to prevent the social problems.

As one of the famous issues in recognition, crowd aggregation prediction  (Chengcai & Ruigang, 2020; Hehe et al., 2020) brings researchers a great challenge. We believe the crowd aggregation prediction should possess high accuracy and stability while facing high crowding, severe occlusion, lousy weather, various scale and crowd heterogeneity. The strategy of people counting is to conduct the regression of the density map  (Huijun et al., 2022; Xiaolong, 2022) for crowds, which is relatively more straightforward than the final task of semantic segmentation for pixel-by-pixel classification, *e.g.*, multi-colunm convolutional neural network (MCNN) (Zhang, Zhou & Chen, 2016) and fully convolutional networks (FCN) (Yifan et al., 2023). The MCNN model is less than 1M, while the FCN model is over 100M. The work is based on convolutional neural networks, so the two fundamental principles must be met. Firstly, the larger the model, the more profound the number of layers is, and the better effect. Secondly, if the model has deeper layers, it will be more possible to happen gradient explosion and gradient disappearance, which means that the training of the model will collapse. Therefore, to follow the inspiration from the perspectives above, as deep as the possible network is adopted. To make the gradient disappear and gradient explosion does not occur in the process of network deepening, the residual connection structure is selected based on ResNet (He et al., 2023).

Several types of research suggest that the convolution layer with the large kernel can result in a better receptive field, which means that the model can achieve more details from the larger region through feedforward together. However, other studies have shown that two 3 × 3 kernels can represent the same nonlinear as a 5 × 5 kernel in the same convolution layer. Thus, we can employ fewer parameters in a model. Then, we can make an easy choice: one is we can achieve a better receptive field and keep the original performance, and another is we can employ fewer parameters in the model and lose a little efficiency. Zhang, Shi & Chen (2018) mentions that they can achieve the effect of 7 × 7 convolution kernels by superimposing two 1 × 7 and 7 × 1 convolution kernels and can cost less computation for the same model. Therefore, we take this idea as a reference and superimposed two groups of 1 × 9 and 9 × 1 convolution kernels to achieve a broader receptive field by replacing the kernel. We calculate coordinates through a network of pedestrians to extract three crowd state features drawn on each eigenvalue analysis. Furthermore, we can employ these scenes and feed them into a proposed double-LSTM model to generate captions for news text collection. We can demonstrate from the experiment results analysis that three kinds of features can respond to the crowd scattered when abnormal change and fluctuation, which can judge the scattered exception very well.

## Related Work

In recent years, CNN has significantly progressed and is applied in face recognition, object detection, pedestrian re-recognition and other computer vision studies. This makes researchers more confident in choosing CNN for crowd-density computing and population statistics (Sang et al., 2019). The Alexnet (Ji, Haitao & Zhuo, 2022) network can be used to study population counting and one study replaced the fully connecting layers containing 4,096 neurons with a layer containing only one neuron (da Silva, Bressan & Goncalves, 2019). However, this method has a defect: it can only estimate the population and cannot achieve the density profile for the crowds in an image. Therefore, Fu et al. (2015) divide the crowd density into five ranks. Then, they employ multi-scale CNN to classify the crowd density level. Zhang et al. (2015) find that the model’s accuracy will decrease significantly when the crowd counting model trained from the fixed scene is applied to the new scene. Aiming at addressing these issues, the authors propose a cross-scene crowd-counting framework, which can adapt well to new scenes. Since 2016, Zhang, Zhou & Chen (2016) have proposed a multi-column strategy for CNN named NN, which indicates the filter structure with three columns of different sizes. This method adapts itself to accommodate the different sizes of the human head because of the resolution and perspective. But, this method is too complicated, which can cost too many parameters and train the model in a seriously hard solution. Sam, Surya & Babu (2017) propose switching the CNN network. They apply the regressor to generate density maps consisting of some CNNs with various kernel sizes. Then, an image goes through a classification model to choose the optimal regressor of CNN, and the obtained result becomes the final result. Zhang, Shi & Chen (2018) propose an adaptive scaling CNN, SaCNN. This made a breakthrough in 2017, and the crowd-counting performance is greatly improved. In SaCNN, they optimize the kernel by the geometric adaptive Gaussian to produce a density map of high-quality as ground truth. Besides, they also employ the density map and loss function for sharing optimization. In 2018, Li, Zhang & Chen (2018) proposed the CSRNet to identify scenes of high density. So that they can obtain the counting results of the crowd and density maps which are high-quality; furthermore, they regard the CNN as the front to get the 2D features and regard the void convolution for the back ending to expand sensing regions and to replace the pooling layer, achieving the current optimal effect. Cao et al. (2018) propose a novel codec network, SANet, which combines Euclidean function and local consistency to construct the novel loss.

## Proposed Method

**Figure 1:**
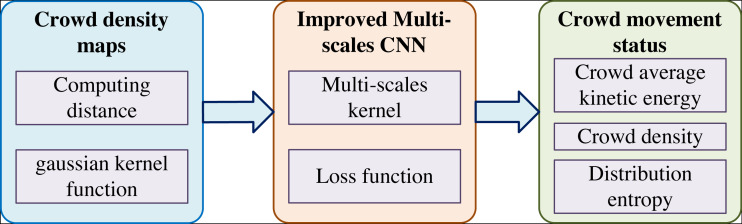
The flow chart of our method. Multi-scale CNN is mainly used to estimate the density of different crowds and the population in images from different angles.

Multi-scale CNN is mainly used to estimate the density of different crowds and the population in images from different angles. Large, medium and small convolutional layers carry out the feature extraction and recognition of images from various distances and angles. The steps of crowd counting in this network are as follows: (1) crowd density map drawing, (2) improved multi-scale convolutional neural network, and (3) crowd motion state value analysis, as shown in Fig. 1.

### Computation of density maps for crowds

The density map of crowds can better and intuitively explain the profile of the crowd. The density map of crowds is drawn by labeling the pedestrian head, replacing the kernel with the geometric adaptive Gaussian to predict the head size of the pedestrian and converting it into density maps, which are then input into the network for training. Assume that the coordinate position of the head center from a pedestrian in an image locates at a point *x*_*i*_, the crowd image with the centers of *N* pedestrian’s heads in the picture is represented as follows:

(1) \begin{eqnarray*}H \left( x \right) =\sum _{i=1}^{N}\delta \left( x-{x}_{i} \right) .\end{eqnarray*}

The geometric adaptive Gaussian kernel function ${G}_{{\sigma }_{i}} \left( x \right) $ is used to calculate the density map *F*, which can be present in the following formula:

(2) \begin{eqnarray*}F \left( x \right) =\sum _{i=1}^{N}\delta \left( x-{x}_{i} \right) \times {G}_{{\sigma }_{i}} \left( x \right) \end{eqnarray*}

where ${\sigma }_{i}=\beta \overline{{d}^{i}}$ and the average distance *x*_*i*_ from the *m* heads of the nearest pedestrian are ${d}^{i}= \frac{1}{m} {\mathop{\sum }\nolimits }_{j=1}^{m}{d}_{j}^{i}$. The simulation results show that the effect is the best at *β* = 0.3.

### Improved multi-scales, CNN

**Figure 2:**
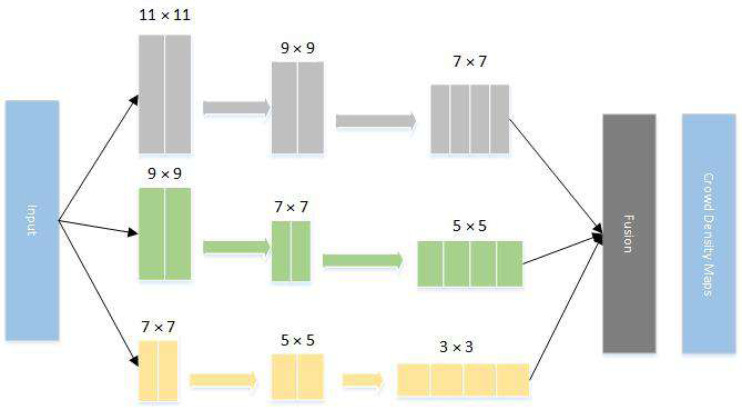
Improved MCNN network structure diagram. The main idea of multi-scale CNN is to fuse the data obtained from the three networks and then extract features to achieve better results.

The main idea of multi-scale CNN is to fuse the data obtained from the three networks and then extract features to achieve better results. Considering the perspective, the network sets up three kinds of networks: large, medium and small. The improved convolution kernels are: the size of *L* column convolution kernels are $ \left[ \left( 11\times 11 \right) , \left( 9\times 9 \right) , \left( 7\times 7 \right) \right] $, *M* column convolution kernels are $ \left[ \left( 9\times 9 \right) , \left( 7\times 7 \right) , \left( 5\times 5 \right) \right] $ and *S* column convolution kernels are $ \left[ \left( 7\times 7 \right) , \left( 5\times 5 \right) , \left( 3\times 3 \right) \right] $. After the images are fed into the three networks, the output data are merged for the density map. The structure diagram of the network is shown in Fig. 2.

The loss function adopted by MCNN network is shown as follows:

(3) \begin{eqnarray*}L \left( \Phi \right) = \frac{1}{2N} \sum _{i=1}^{N}{ \left\| F \left( {X}_{i},\Phi \right) -{F}_{i} \right\| }_{2}^{2}\end{eqnarray*}

where Φ denotes the parameters that can be continuously trained in MCNN, *N* represents the counting of entire images in the training phase, *X*_*i*_ represents the *i* − *the* input, *F*_*i*_ indicates the actual density map extracted from the *i* − *the* image, $F \left( {X}_{i},\Phi \right) $ is the corresponding density map and *L* represents the loss function between the calculated density map and the ground truth.

The following two general evaluation values are selected to evaluate the performance for choosing the training model: MAE and MSE. The calculation formula for the two evaluation values is as follows:

(4) \begin{eqnarray*}MAE= \frac{1}{N} \sum _{i=1}^{N} \left\vert {z}_{i}-{\tilde {z}}_{i} \right\vert \end{eqnarray*}

(5) \begin{eqnarray*}MSE=\sqrt{ \frac{1}{N} \sum _{i=1}^{N}{ \left( {z}_{i}-{\tilde {z}}_{i} \right) }^{2}}.\end{eqnarray*}

### Crowd movement status

The above content introduces the network used in this article and the improvement part in detail. The purpose of using this network structure is as follows: (1) generating crowd density map by network prediction. (2) The head coordinate points of individual pedestrians in each surveillance video frame are predicted through the network. (3) The pedestrian coordinate points are extracted to calculate the characteristic values of the crowd motion state.

The crowd behavior is in a state of constant motion. In this article, the dynamic eigenvalue changes are used to determine whether abnormal crowd sudden dispersion behavior occurs, which are defined as the following three crowd movement state eigenvalues. The first is the average kinetic energy of the crowds. In the monitoring scenario, the average kinetic energy of the crowds will increase when sudden crowd dispersions occur. The second is the crowd density value. Due to the limited monitoring range under the surveillance video, when the crowd breaks out, the crowd will leave the surveillance video and the crowd density value will decrease accordingly. The third is the crowd distribution entropy. The crowd distribution in the monitoring area will change when crowd dispersion occurs. As the crowd dispersion proceeds, the crowd distribution entropy will become larger and more prominent with the crowd dispersion.

(1) Average kinetic energy of the crowd.

Kinetic energy is simply the energy of an object due to its motion. The traditional method determines the feature points by extracting the corner information of the foreground object and then calculates the crowd kinetic energy according to the motion vector of the identified feature points. In this article, the kinetic energy refers to the average kinetic energy from crowds in monitoring areas, and the position $ \left( {x}_{0},{y}_{0} \right) $ of the center point of the image is selected for each frame of the image.

As the reference point, the average distance *s*_*i*_ Between the network predicted coordinate points in the *i*th frame and the reference point is calculated. Assume that *j* pedestrian heads coordinate points are anticipated in the *i* − *the* frame. Then, the average distance *s*_*i*+1_ of the (*i* + 1)-th frame is calculated and the speed of the first frame is calculated according to the frame rate. Therefore, we can calculate the average kinetic energy of crowds. The formula is presented as follows:

(6) \begin{eqnarray*}{s}_{i}= \frac{1}{N} \sum _{i=1}^{N}\sqrt{{ \left( {x}_{j}-{x}_{0} \right) }^{2}+{ \left( {y}_{j}-{y}_{0} \right) }^{2}}\end{eqnarray*}

(7) \begin{eqnarray*}{E}_{avg}= \frac{1}{2} m{ \left( \frac{{s}_{i+1}-{s}_{i}}{t} \right) }^{2}\end{eqnarray*}

where *E*_*avg*_ Represents the average kinetic energy of crowds, *t* is the time of a single frame and *s*_*i*_ is the average distance of the *i*th frame. Since the size of moving objects in the motion scene is similar, so *m* = 1.

(2) Crowd density value.

The crowd density value is used to judge the proportion of the corresponding area occupied by the crowd in a frame. The improved MCNN can generate the density map and simultaneously calculate the predicted number of people in the frame. The specific crowd density value is shown as follows:

(8) \begin{eqnarray*}{D}_{i}= \frac{\lambda {N}_{i}}{{S}_{i}} \end{eqnarray*}

where we regard the number of people predicted in the *i*th frame as *N*_*i*_, *λ* denotes the correction factor of the counting of people in an image and *S*_*i*_ Indicates the image area in the *i*th frame. We set *S*_*i*_ = 1 to simplify the computation.

(3) Crowd distribution entropy.

Entropy is used to calculate and estimate the amount of information, reflecting random time’s uncertainty. In this article, the idea of information entropy is used to calculate the distribution of the crowd. If the crowd is scattered, the entropy of the crowd distribution will increase, and vice versa. Firstly, the coordinates are normalized to make them between $ \left[ -1,1 \right] $. Then, the area is divided into 20 continuous small regions on average, which are $ \left[ \left( -1,-0.9 \right) ,\ldots , \left( 0.9,1 \right) \right] $. The crowd distribution entropy is calculated using the following formula:

(9) \begin{eqnarray*}S \left( n \right) =-\sum _{i=1}^{k}{p}_{i}\log \nolimits 2~{p}_{i}\end{eqnarray*}

(10) \begin{eqnarray*}{p}_{i}= \frac{\text{count} \left( {y}_{k} \right) }{\sum _{i=1}^{k}\text{count} \left( {y}_{k} \right) } .\end{eqnarray*}

### Scene captioning model

**Figure 3:**
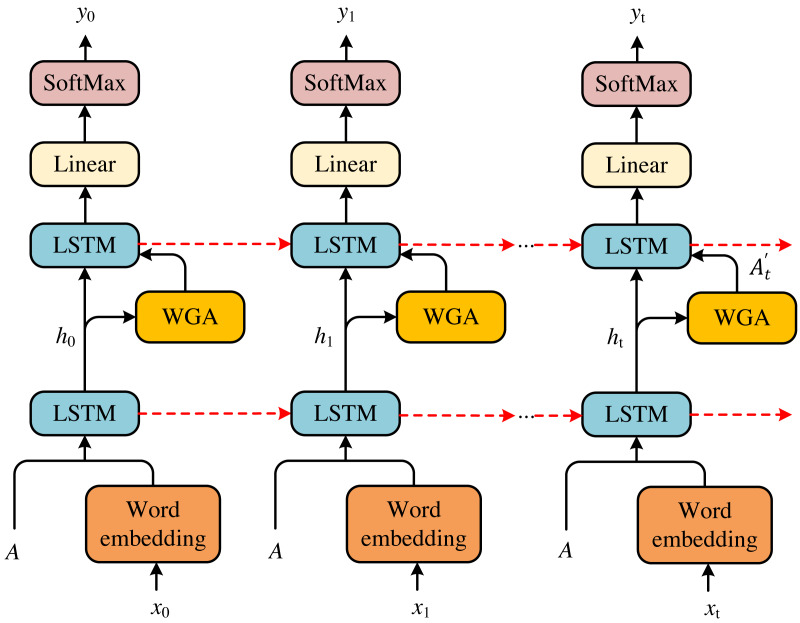
Double-LSTM structure diagram. According to the models above, we can achieve the scene about a happening event in the environment of health emergence. To accomplish the news text about this event, we propose a double-LSTM to generate captions for the scene. The model is presented in Fig. 3.

According to the models above, we can achieve the scene about a happening event in the environment of health emergence. To accomplish the news text about this event, we propose a double-LSTM to generate captions for the scene. The model is presented in Fig. 3.

Given the scene information *I*, we first feed it into the word generation attention (WGA) to attend to the previous information *h*_*t*−1_. Before computing attention, we must apply the word *x*_*t*−1_ and the cell features *A* to achieve the *h*_*t*−1_. Then, we feed *h*_*t*−1_ and the results of attention ${h}_{t}^{a}$ into the language LSTM to obtain the predicted word *y*_*t*_. Therefore, we can achieve a whole caption $ \left\{ {y}_{0},{y}_{1},\ldots ,{y}_{t} \right\} $ for the current scene to get the news text. The entire process can be formulated as follows:

(11) \begin{eqnarray*}{h}_{t-1}={\text{LSTM}}^{a} \left[ A,{x}_{t-1} \right] \end{eqnarray*}

(12) \begin{eqnarray*}{h}_{t-1}^{l}={\text{LSTM}}^{l} \left[ {h}_{t-1},{h}_{t}^{a} \right] \end{eqnarray*}

(13) \begin{eqnarray*}{y}_{t}=\text{Softmax} \left( {h}_{t-1}^{l}W+b \right) \end{eqnarray*}

## Experiments

### Dataset and implement details

This experiment is carried out on an ordinary PC with AMD Ryzen 5 3600 CPU, 3.60ghz, 16G memory, RTX 2070 SUPER graphics card, 8G. The improved MCNN network is built in Anaconda3 + Pytorch1.3.1 + cuda10.1 + cudnn7.5.1 + Python3.7.1 environment for training and testing and the final crowd state eigenvalue analysis is completed in Python3.8 environment.

All population estimation experiments are performed on the ShanghaiTech dataset. There are 1,198 labeled images in this dataset, which includes two different parts which are Part_A and Part_B. In Part_B, the images are sparser than those in Part_A. ShanghaiTech dataset is first established in Zhang, Zhou & Chen (2016), where they donate 300 images to train the model in Part_A and 182 images are regarded as the samples to test. Part_B includes 400 training images and 316 testing images. We use the videos of abnormal crowd activity located indoors in the dataset UMN of the University of Minnesota to predict the trend of crowd aggregation. In addition, we apply the MSCOCO to evaluate the scene captioning model for news text collection.

### The results and comparison

To evaluate our method, we compare our method with MCNN, MSCNN, CMTL, Switching CNN, SaCNN, TDF-CNN and CSNet models. We present the experimental results in Table 1.

We can conclude that compared with the MCNN model in the ShanghaiTech dataset, the MAE and MSE of our model decrease by 22.0 and 35.7 on Part_A and decrease by around 16.5 and 39.7 on Part_B, respectively. Compared with CMTL, MAE and MSE on Part_A decrease 26.2 and 14.7 and MAE and MSE on Part_B decrease 9.8 and 17.8, respectively. In addition, compared with MSCNN, Switching CNN, SaCNN and TDF-CNN, our method has an absolute leading position on Part_A and comprehensively outperforms the above techniques on Part_B. However, when comparing with TDF-CNN, our approach lags slightly behind on Part_A and beats TDF-CNN on Part_B, which indicates that our method possesses wider affection than TDF-CNN in the scenario while testing Less foot traffic. In summary, compared with other competitors, our approach can better predict the crowd gathering in different scenes. It can fully provide the necessary predictive information for news text collection and social problem prevention.

### Experimental analysis of crowd aggregation

The eigenvalue calculation in this article is based on the video of indoor abnormal crowd activity in the UMN dataset of the University of Minnesota. The detection and analysis of two weird videos, random burst and same-direction burst, are carried out, respectively. The crowd’s density and the crowd’s distribution entropy, we distinguish the irregular and the same direction of the weird videos.

**Table 1:** Comparison with the different models on the ShanghaiTech dataset. To evaluate our method, we compare our method with MCNN, MSCNN, CMTL, Switching CNN, SaCNN, TDF-CNN and CSNet models.

Methods	ShanghaiTech Part A		ShanghaiTech Part B	
	MAE	MSE	MAE	MSE
Zhang, Zhou & Chen (2016)	95.5	165.6	24.2	52.1
MSCNN	93.1	112.3	15.4	28.5
CMTL	99.7	142.6	18.5	30.2
Sam, Surya & Babu (2017)	88.3	129.4	22.2	28.1
Zhang, Zhou & Chen (2016)	76.2	139.1	17.5	24.3
TDF-CNN	104.3	139.4	27.8	29.1
CSNet	72.3	117.4	9.1	17.2
Our method	73.5	127.9	7.7	12.4

**Figure 4:**
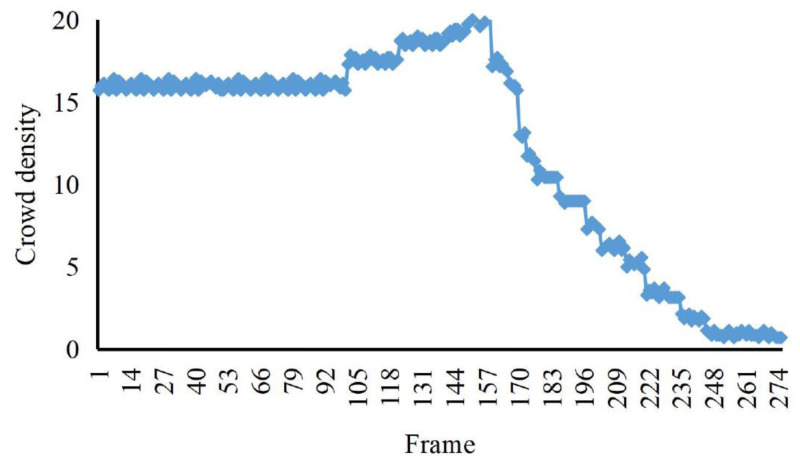
The crowd density eigenvalues of irregular sudden increase abnormalities. The eigenvalue calculation in this article is based on the video of indoor abnormal crowd activity in the UMN dataset of the University of Minnesota.

**Figure 5:**
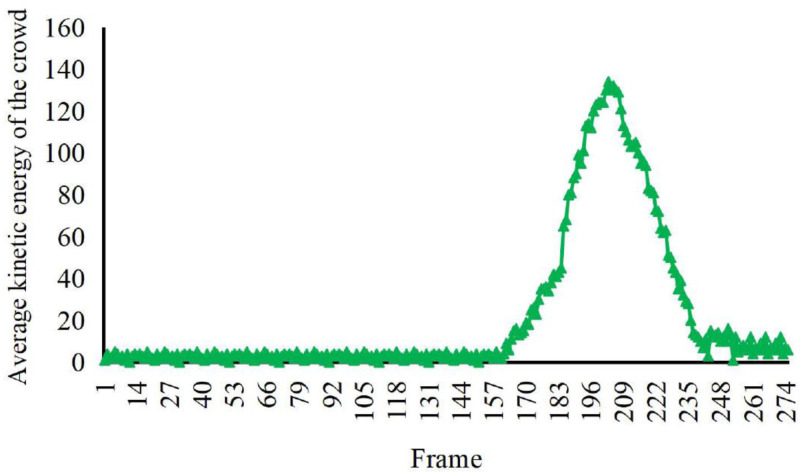
Characteristic values of average kinetic energy of the crowd with irregular spurt anomaly. The detection and analysis of two weird videos, random burst and same-direction burst, are carried out, respectively. The crowd’s density and the crowd’s distribution entropy, we distinguish the irregular and the same direction of the weird videos.

**Figure 6:**
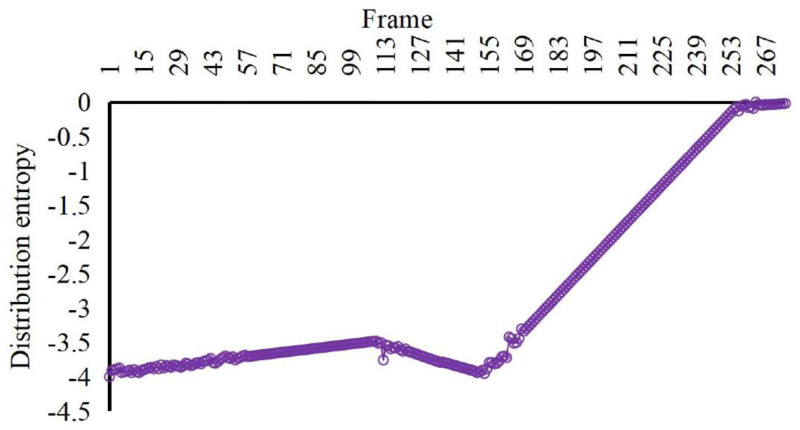
Characteristic values of distribution entropy of the crowd with irregular spurt anomaly. According to the corresponding frames in the video, it can find that the inflection points of the change of the eigenvalues of the three states corresponded to the moment when the crowd started to disperse abruptly in the video.

According to the comparison in Figs. 4, 5 and 6, at about 170th frame, the three eigenvalues all begin to change significantly, the kinetic energy and crowd distribution entropy begin to increase sharply and the crowd density gradually decreases. According to the corresponding frames in the video, it can find that the inflection points of the change of the eigenvalues of the three states corresponded to the moment when the crowd started to disperse abruptly in the video. At this moment, the crowd suddenly disperses and starts to run and the kinetic energy and distribution entropy gradually increase. The crowd density gradually decreases as pedestrians run out of the monitoring area. We can find from the figure that the average kinetic energy of the first increase then decreases slowly until about 245th frame returns to the initial values of kinetic energy. The main reason for this anomaly which is different from the former, is pedestrians do not entirely run out of the monitor screen, keep steady and stop running slowly back to walking posture after the 245th frame of the video. Because the pedestrians in the surveillance video are fewer, it can be seen that the value of distribution entropy also starts to level off at around 250th frame.

### Results of scene captioning

**Figure 7:**
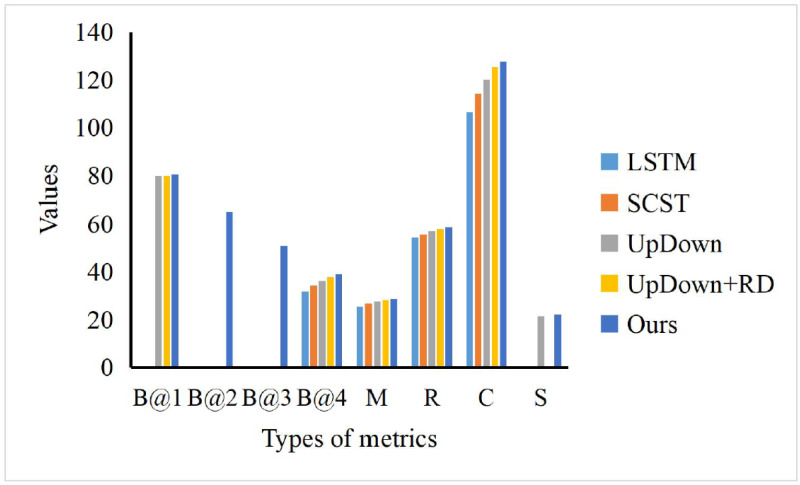
The comparison of our model and others. To evaluate our scene captioning model, we choose the LSTM, SCST, UpDown and UpDown+RD to compare with our method. As shown in Fig. 7, where B@N, M, R, C and S denote the evaluation metrics of BLEU-N, METEOR, Cider and Spice, respectively, our model can achieve the best performance while comparing with other models, especially in the term of Cider scores (C).

To evaluate our scene captioning model, we choose the LSTM, SCST, UpDown and UpDown+RD to compare with our method. As shown in Fig. 7, where B@N, M, R, C and S denote the evaluation metrics of BLEU-N, METEOR, Cider and Spice, respectively, our model can achieve the best performance while comparing with other models, especially in the term of Cider scores (C). In the term of BLEU-1 (B@1), we can find that our model surpasses others just a little. However, our model can obtain the obvious advantages, which means that our model can be fed with the scene information and generate accurate text information for the happening event, which can be helpful for the news text collection.

## Conclusion

We believe that most of the news and social issues in these catastrophes are essentially related to people when considering news gathering and social concerns in public health emergencies. We can determine the population density and forecast the emergence of news events and societal issues by continuously tracking the change in the population. Additionally, we can keep processing the scene to create the captions required. This study uses a number estimate approach to count the number of real-time modifications. We employ the enhanced multi-scale convolutional neural network to track each frame of the video and consider the number of people, which can be examined to determine the pedestrian’s coordinates for the location and lay the groundwork for further movement in the calculation. The number of individuals estimated in this research for small and medium-density populations is a better predictor since, the MCNN has been enhanced. We compute the coordinates of three crowd state features based on each eigenvalue analysis using a network of pedestrians. Finally, using the suggested double-LSTM, we apply the scenario predicted above to produce the news text. The study of the experiment’s findings reveals three categories of characteristics that, in the presence of an abnormal change or fluctuation, can lead the crowd to disperse and which can be utilized to determine the scattered exception exceptionally precisely. Additionally, our double-LSTM model can provide the text of the current occurrence in the scene for news text gathering. In the future, we will study the expression of crowd action content and directly identify it to improve the accuracy of press release generation.

##  Supplemental Information

10.7717/peerj-cs.1283/supp-1 Supplemental Information 1 The code for this article

## Additional Information and Declarations

### Data Availability

The following information was supplied regarding data availability: The dode is available in the Supplemental File. The data is available at Zenodo: Xiaoling Zhou. (2023). A crowd clustering prediction and captioning technique for public health emergencies (Version V2) [Data set]. Zenodo. https://doi.org/10.5281/zenodo.7601883
