# Supplementary material for: A crowd clustering prediction and captioning technique for public health emergencies
Source: PeerJ Comput Sci. 2023 May 4;9:e1283. doi: 10.7717/peerj-cs.1283 (PMC10280499; doi:10.7717/peerj-cs.1283)
Supplement: Supplemental Information 1 [file peerj-cs-09-1283-s001.zip › ┤·┬δ/scene captioning/vis/index.html]

neuraltalk2 results visualization
